# Complete agenesis of pericardium in a young asymptomatic woman

**DOI:** 10.1016/j.radcr.2024.04.020

**Published:** 2024-05-10

**Authors:** Giuseppe Maria Andrea D'Arma, Domenico Riccardo Rosario Chieppa, Valentina Forte, Federica Masino, Francesco Bartolomucci, Giuseppe Guglielmi

**Affiliations:** a Department of Clinical and Experimental Medicine, Foggia University School of Medicine, Viale L. Pinto 1, 71122 Foggia (FG), Italy; b Cardiology Unit, “Bonomo” Hospital, Viale Istria 1, 76123, Andria (BT), Italy; c Radiology Unit, “IRCCS Giovanni Paolo II” Hospital, Viale Orazio Flacco 65, 70124, Bari, Italy; d Radiology Unit, “Dimiccoli” Hospital, Viale Ippocrate 15, 70051, Barletta (BT), Italy; e Radiology Unit, “IRCCS Casa Sollievo della Sofferenza” Hospital, Viale Cappuccini 1,71013, San Giovanni Rotondo (FG), Italy

**Keywords:** Pericardial agenesis, Heart congenital defect, Cardiac magnetic resonance, Cardiology, Diagnostic imaging, Pericardium

## Abstract

Agenesis of pericardium is a rare finding resulting from alterations during embryologic formation. It is a congenital cardiac anomaly commonly asymptomatic. Cardiac magnetic resonance is actually considered the gold standard for diagnosis of pericardium agenesis. This report details the case of a 24-year-old woman who came to our clinic.

## Introduction

Pericardium is a vascular fibro-serous sac composed of an outer fibrous layer and an inner double layered serous membrane with parietal and visceral layers. The visceral layer is strictly bound to the heart surface and is separated from the second one by a virtual space filled by pericardial fluid (15-50 mL normally). The primary function of pericardium is to give a correct position to heart and to prevent potential torsion of large vessel. Another function is to reduce the friction during heart beats [[1], [2], [3]]. Pericardial defects are relatively rare disorders that could be distinguished as acquired or congenital. The first one is caused by surgery (pericardiectomy) in order to solve constrictive or recurrent pericarditis.

Congenital defects are rare condition, with incidence of <1 in 10,000 cases. These defects are predominantly found in males, with a male-to-female ratio of 3:1 [4]. Agenesis of pericardium can be characterized by location and classified as incomplete or partial and complete. More than 70% of cases are represented by complete left agenesis of pericardium, which is usually asymptomatic. Complete bilateral agenesis is reported for 9%, right-side defects figure for remaining portion [2,[5], [6], [7]]. We report the case of a 24-year-old woman who was referred to our clinic.

## Case report

A 24-year-old patient was referred to our institution. She had no significant medical history, cardiovascular risk factors, or family history of sudden-cardiac-death. Physical exam was non-specific, without particular findings. ECG revealed right axis deviation and bradycardia. Echocardiography was not diagnostic due to inability to obtain adequate acoustic windows, but the apical one showed a marked left displacement of heart. In order to further investigate was requested a diagnostic study by cardiac magnetic resonance (CMR). Previously she performed blood test that showed no significant anomalies.

**Fig. 1:**
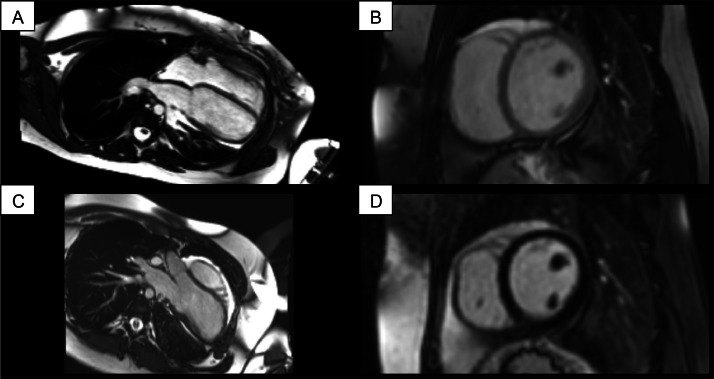
(A-C) ECG-gated cine-SSFP sequences, 4 chambers, mid-ventricular short axis, 3 chambers. Abnormal heart position with left ventricular chamber dislocated to the left and apex directed posteriorly. It is possible to see the direct contact of epicardial fat with heart and mediastinal pleura. Pericardium cannot be observed; (D) ECG-gated PSIR. No evidence of LGE. Pericardium cannot be observed.

The CMR, using contrast media with Late Gadolinium Enhancement (LGE), SSFP cine sequences, and T2-STIR sequences, showed normal volumes and functions for both ventricles and atria. No oedema or fibrosis was demonstrated with proper sequences. Furthermore, SSFP cine sequences revealed a heart abnormal position inside mediastinum, with left ventricular chamber dislocated to the left, with apex directed posteriorly. Moreover, no evidence of pericardial layers was demonstrated in all sequences, with direct contact of epicardial fat with heart and mediastinal pleura. All these findings were relevant for the diagnosis of complete pericardial agenesis (Fig. 1).

## Discussion

Congenital anomalies of pericardium are rare cardiac anomalies which primarily include pericardial cysts, diverticula, and agenesis of pericardium. Among these, congenital agenesis of pericardium is the most uncommon. It is usually identified incidentally in autopsies or surgical procedures, with an approximately prevalence of 0.002%-0.004%, but actually thanks to development of numerous and advanced cardiac imaging modalities (CMR-TC-Echocardiography) a rising prevalence is seen [[7], [8], [9], [10], [11]]. Congenital agenesis of pericardium results from alterations during embryologic formation of pericardium. Its pathogenesis is multi-factorial, but it seems to depend on premature atrophy of the left common cardinal vein [12]. It causes a reduced blood supply to left pleuro-pericardial fold, causing its abnormal formation and closure [3,7,13].

The disease is more common in males with a ratio M:F of 3:1 [2,4]. This congenital pathology can be characterized by location and classified as incomplete or partial and complete. It is possible to distinguish 6 types of congenital agenesis of pericardium: complete bilateral agenesis, left complete and partial agenesis, right complete and partial agenesis and finally a diaphragmatic form. More than 70% of cases are represented by complete left agenesis of pericardium [2,14]. About 30%-50% of cases are associated with other cardiac anomalies, like patent ductus arteriosus, mitral valve stenosis, bronchogenic cyst, ventricular septal defect as in the case of tetralogy of Fallot. Diaphragmatic form usually comes together with diaphragmatic hernia [2,[5], [6], [7]].

Presentation is usually variable, and complete forms are characterized for the absence of symptoms. However, it is also possible to see a non-specific symptom like dyspnea, fatigue, sometimes heart failure, pericarditis and recurrent pulmonary infections. Also, arrhythmias and sudden cardiac death are reported in literature [1,2,7]. The most common symptom is chest pain. The underlying mechanism has not fully explained, but the pain appears to have angina-like nature. A possible explanation is the risk of extrinsic coronary compression due to a fibrous pericardial rim, with significant obstruction to blood flow. Presence of a pericardial defect should be suspected specially in presence of migratory obstruction of coronary tree in absence of significant abnormalities or atherosclerotic disease. Other mechanisms are the torsion of great vessels and possible presence of adhesion between heart and pleura. Moreover, the complete bilateral agenesis promotes heart dislocation and consequent twist of great vessels and possible tricuspid insufficiency [15,16]. Agenesis of pericardium, due to hyper-mobility of heart, could be responsible of complications as cardiac herniation and strangulation (specially for left atrial appendage) and high risk of fatal arrhythmias. Diagnosis is usually difficult because of non-specific signs and symptoms. Cardiac imaging plays an important role, specifically echocardiography, CCT, and CMR. The first one is economic, relatively simple, but has not an elevated specify and sensitivity for this pathology [6]. For this reason, CCT and CMR emerged as first choice for diagnosis of pericardium pathologies. Compared to CCT, CMR is more accurate, without need of X-rays emission, and offer a better overview of mediastinum with the best combination of contrast, spatial and temporal resolution. For this reason, CMR is considered the gold standard for diagnosis of pericardium pathologies [2,[9], [10], [11]].

**Fig. 2:**
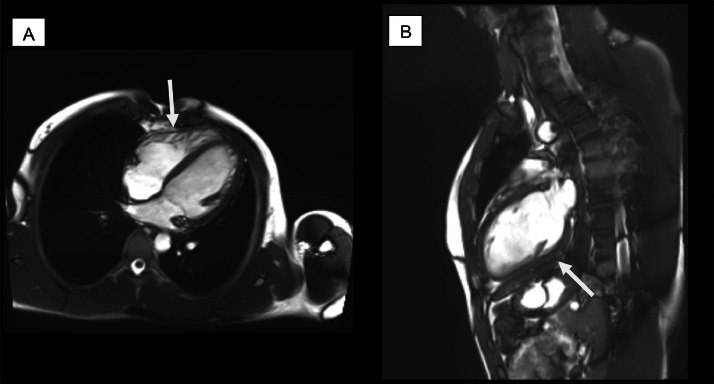
(A and B) ECG-gated cine SSFP sequences, 4 chamber and 2 chamber. Normal pericardium appears as a dark curvilinear structure that following the profile of heart (white arrows) and is surrounded by fat tissue, characterized by a high signal intensity. Normal pericardium thickness is less than 2 mm. These images are of a patient who came to our clinic and gave informed consent.

The normal pericardium appears on MR images as a dark curvilinear structure that following the profile of heart, that has medium signal intensity in T1 and T2 weighted spin-echo, and is surrounded by fat tissue, characterized by a high signal intensity (Fig. 2).

Normal pericardium thickness is less than 2mm on CMR. It develops pericardial sinus and recesses that usually contain some fluid [10,11].

On CMR the diagnosis of agenesis of pericardium depends on the type of this pathology. In fact, it is relatively simple for right agenesis, because it is easier to see the absence of this structure along the right free wall. More difficult is to diagnose left agenesis of pericardium, due to the increased difficulty in distinguishing pericardium from left ventricle. Rather than directly viewing pericardium in these cases some indirect signs are helpful, like 1) excessive levo-rotation with posterior displacement of heart; 2) cardiac indentation at the location of defect; 3) interposition of lung tissue where pericardium is absent; 4) herniation of cardiac tissue [2,4,10,11]. Moreover 33% of cases are associated with other congenital defects like bronchogenic cysts, ventricular septal defect, patent ductus arteriosus or diaphragmatic herniation.

New quantitative parameters were proposed by Macaione et Al. [17] in order to increase the sensitivity of CMR. The first one was a clockwise rotation of heart with a cut-off of 60°.It is measured in the end-diastolic phase, as the angle between an imaginary line tracked from anterior to posterior wall of vertebral body and left ventricle axis and could help to distinguish left complete agenesis of pericardium to the right ventricular overload (another frequent cause of clockwise rotation). The second proposed parameter was a Whole-Heart-Volume-Change (WHVC) >13%, represented by the variation, expresses as a percentage, of the heart volume between systole and diastole. These cut-off values appear to be reproducible criteria for diagnosis of left complete agenesis of pericardium with CMR.

Actually, due to the lack of controlled studies, there are not guidelines for management of agenesis of pericardium. Recommendations are principally based on clinical reports and evidence. Because complete absence of pericardium is commonly asymptomatic an intervention is not needed. Instead for partial agenesis surgery is frequently needed when patients develop symptoms or have high risk of cardiac structure herniation or strangulation and incarceration. Surgical options include pericardioplasty or pericardiectomy with or without valve replacement if needed [12]. Left appendage strangulation is treated with excision and closure with patch [2,7,9,14,16]. In our case, due to the absence of symptoms there was no need of treatment.

## Conclusion

This report presents a case of complete pericardial agenesis. As most reported in the literature, our patient was asymptomatic and required no treatment. Nevertheless, diagnosis is important, especially in case of symptomatic patients, and to get there a combination of different cardiac imaging modalities could be used. We focused on CMR, with qualitative and future quantitative parameters, that actually is considered the gold-standard.

## Ethic committee

No ethical approval is required.

## Authors contribution

The authors confirm contribution to the paper as follows: study conception and design: DGMA. Author CDRR. Author; data collection: CDRR. Author BF. Author; analysis and interpretation of results: CDRR. Author FV. Author; draft manuscript preparation: DGMA. Author, MF. Author. GG. Author. All authors reviewed the results and approved the final version of the manuscript.

## Patient consent

Complete written informed consent was obtained from the patient for the publication of this study and accompanying images.
